# Replication Origin Selection Regulates the Distribution of Meiotic Recombination

**DOI:** 10.1016/j.molcel.2014.01.022

**Published:** 2014-02-20

**Authors:** Pei-Yun Jenny Wu, Paul Nurse

**Affiliations:** 1 Institute of Genetics and Development of Rennes, CNRS UMR 6290, 2 Avenue du Pr. Léon Bernard, 35043 Rennes, France; 2 The Rockefeller University, 1230 York Avenue, New York, NY 10021, USA; 3 The Francis Crick Institute, 215 Euston Road, London NW12BE, UK

## Abstract

The program of DNA replication, defined by the temporal and spatial pattern of origin activation, is altered during development and in cancers. However, whether changes in origin usage play a role in regulating specific biological processes remains unknown. We investigated the consequences of modifying origin selection on meiosis in fission yeast. Genome-wide changes in the replication program of premeiotic S phase do not affect meiotic progression, indicating that meiosis neither activates nor requires a particular origin pattern. In contrast, local changes in origin efficiencies between different replication programs lead to changes in Rad51 recombination factor binding and recombination frequencies in these domains. We observed similar results for Rad51 when changes in efficiencies were generated by directly targeting expression of the Cdc45 replication factor. We conclude that origin selection is a key determinant for organizing meiotic recombination, providing evidence that genome-wide modifications in replication program can modulate cellular physiology.

## Highlights

• Premeiotic S phase neither activates nor requires a specific origin usage program
• Origin selection is a key regulator of the distribution of meiotic recombination
• Organization of genome duplication has a functional impact on cellular physiology

## Graphical Abstract

**Figure:**
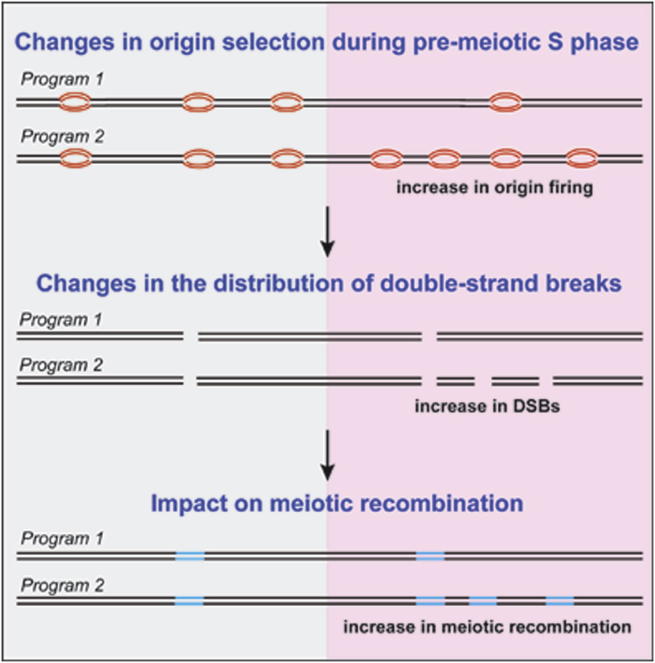

## Teaser

Changes in the pattern of DNA replication are observed during development and in various pathologies, but it is unclear whether they contribute to these processes. Wu and Nurse show, by altering replication origin usage in fission yeast, that origin selection is critical for the organization of meiotic recombination.

## Introduction

Eukaryotic DNA synthesis initiates at origins of replication distributed throughout the genome. The program of DNA replication is defined by the timing of firing of each origin during S phase and the probability of usage of each origin in a population of cells, or origin efficiency. Origin selection is modulated by diverse inputs, including cell-cycle regulation, chromatin modifications, nucleotide levels, and gene transcription (Aladjem, 2007, Méchali, 2010, Gilbert, 2007, Cayrou et al., 2010). Alterations in the pattern of replication have been observed in *Xenopus* and *Drosophila* during development (Méchali, 2010, Hyrien and Méchali, 1993), in differentiating mouse and human cells (Grégoire et al., 2006, Ryba et al., 2010, Hiratani et al., 2008), and in cancers (Amiel et al., 1998, Dotan et al., 2004), but it remains unknown whether undergoing S phase with particular programs of replication has direct consequences on cellular function. A major change in cellular physiology is the transition to the process of meiosis, which ensures the production of gametes for sexual reproduction and the exchange of genome complements. A genome-wide analysis in fission yeast has suggested that there are significant alterations in origin efficiencies between the mitotic and meiotic cell cycles (Heichinger et al., 2006). Therefore, we have investigated the impact of origin selection during premeiotic S phase on the subsequent events in meiotic DNA metabolism, characterized by two rounds of chromosome segregation, including a reductional division and interhomolog recombination (Roeder, 1997).

## Results

### Premeiotic S Phase Neither Requires nor Activates a Particular Program of Replication

**Figure 1:**
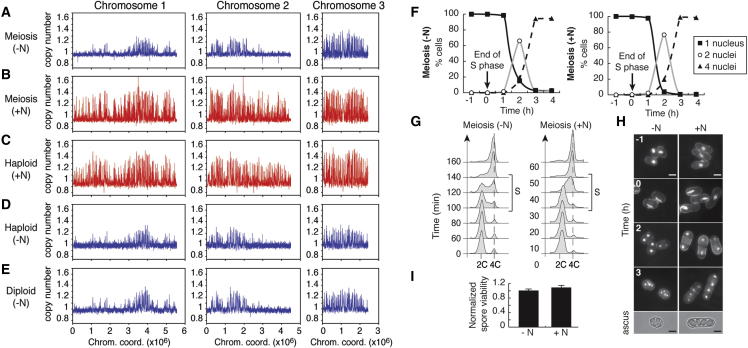
Changes in Replication Origin Selection during Premeiotic S Phase Do Not Affect Overall Meiotic Progression (A) Replication profile in *pat1-114* diploids induced to undergo meiosis after nitrogen starvation. (B) Replication profile in *pat1-114* diploids induced to undergo meiosis when grown continuously in nitrogen-rich medium. (C) Replication profile in haploid cells grown vegetatively in nitrogen-rich medium. (D and E) Replication profiles in haploid (D) and diploid (E) cells that reentered the mitotic cycle after nitrogen starvation. See Figure S1A and Supplemental Experimental Procedures for details of the protocols for (A)–(E). −N: after nitrogen depletion (blue). +N: continuous nitrogen-rich conditions (red). The *pat1-114* mutation allows for induction of meiosis at the restrictive temperature of 34°C. To determine origin efficiencies, cells were treated with hydroxyurea (HU) prior to S phase and harvested at time points when DNA replication is complete in the control samples without HU (data not shown). Genomic DNA was isolated and copy number determined using Agilent 4×44k *S. pombe* arrays. x axis: chromosome coordinates; y axis: copy number. (F) Time course of meiotic progression of *pat-114* diploids induced to undergo meiosis. Heat-fixed cells were stained with DAPI to detect nuclei, and the number of nuclei per cell was counted each hour to determine the kinetics of meiosis I and II after nitrogen depletion (−N) and in continuous nitrogen-rich conditions (+N) (n ≥ 200). The time for the end of premeiotic S phase (t = 0) was determined by flow cytometry (see G). In both cases, most cells have proceeded through meiosis I around 2 hr and meiosis II around 3 hr after the completion of S phase. (G) Length of premeiotic S phase in conditions as in (F). Flow cytometry analysis of cell-cycle progression. t = 0 corresponds to the time of the shift to restrictive temperature (Figure S1A). Duration of DNA replication is indicated by brackets. Premeiotic S phase in continuous nitrogen-rich conditions is approximately half the length of that in cells that have been nitrogen starved. (H) Cytology of meiosis in conditions as in (F). Live cells were stained with Hoechst and Blankophor to visualize nuclei and cell walls. Asci were visualized by DIC. Scale bars, 5 μm. Times are as in (F). The horsetail movement characteristic of meiosis in fission yeast can be seen at t = 0 in both conditions. (I) Spore viability is unaffected by changes in the replication program. Viability of spores in conditions as in (F). Identical numbers of isolated spores (at 10 hr and 8 hr after induction of meiosis for −N and +N, respectively) were plated on rich medium for analysis of viability. Average of 3 independent experiments (n ≥ 200) with SE normalized to the viability after nitrogen starvation.

First, we determined if the characteristic changes in the replication program observed during meiosis (Heichinger et al., 2006) are a consequence of the commitment to meiotic progression or result from the environmental conditions that lead to meiosis. In fission yeast, nitrogen starvation triggers the mating of haploid cells to generate diploids that undergo meiosis and sporulation. We therefore tested the effect of changing the nutritional conditions on the program of origin usage in premeiotic S phase. To this end, we induced meiosis in nitrogen-rich medium by utilizing the well-characterized *pat1-114* temperature-sensitive mutation in diploid fission yeast cells (Iino and Yamamoto, 1985). At permissive temperature, these cells are sensitive to mating pheromones (M-factor in our experiments), which bring about G1 arrest (Davey and Nielsen, 1994). Subsequent shift to restrictive temperature allows synchronous entry into meiosis in the continuous presence of a nitrogen source. We assessed the replication program in these conditions or following a standard nitrogen starvation procedure (Figure S1A available online). To determine origin efficiencies in these experiments, cells were treated with hydroxyurea (HU) prior to the start of S phase, and genomic DNA was analyzed by microarrays for increase in copy number (Heichinger et al., 2006). Our results show that the program of premeiotic replication in cells continuously grown in nitrogen-rich medium (+N) is significantly different from that in cells entering the meiotic cycle after nitrogen starvation (−N), with a considerable increase in the number and efficiency of origins (Figures 1A, 1B, S1B, and S1C). This indicates that the meiotic replication program previously described in fission yeast (Heichinger et al., 2006) is a result of nutritional status rather than commitment to meiosis per se. Furthermore, the patterns of origin usage between mitotically cycling cells and meiotic cells maintained in nitrogen-rich medium are very similar (Figures 1B and 1C). Consistent with this, we also found that after nitrogen starvation, origin selection in vegetatively growing cells is virtually identical to that observed in premeiotic S phase in the same −N conditions (Figures 1A, 1D, and 1E). We conclude that nitrogen availability modulates origin selection and efficiencies in both meiotic and mitotic cycles. Comparison of the gene expression profiles at the start of S phase during mitotic cycles after nitrogen starvation and in nitrogen-rich conditions (based on data from Mata et al., 2002 and Rustici et al., 2004) suggests that the observed alterations in the pattern of origin efficiencies do not reflect differences in the transcription profiles along the chromosomes (data not shown). Importantly, the significant changes in premeiotic origin usage in different nitrogen conditions (Figures 1A and 1B) provide an effective system for assessing the contribution of the replication program to meiotic progression.

**Figure 2:**
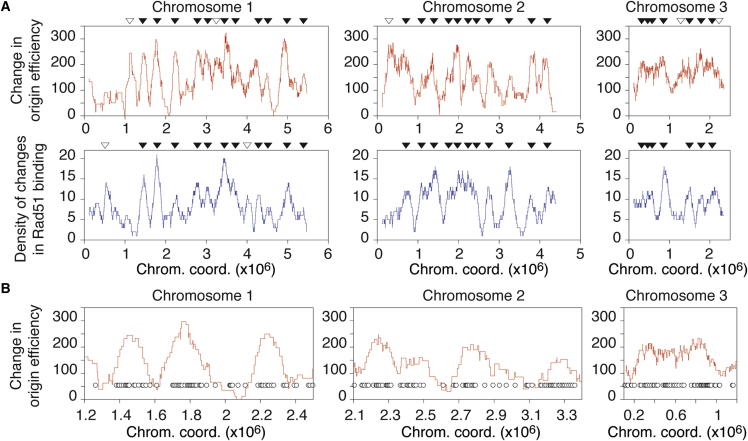
Changes in Origin Efficiencies between Replication Programs Correspond with Changes in Rad51 Binding (A) Genome-wide analysis of changes in origin efficiencies and Rad51 binding. Top panels: alterations in origin efficiencies in premeiotic S phase. The sums of origin efficiencies as in Figure 1 were determined for continuous ∼200 kb windows for premeiotic S phase after nitrogen depletion (−N) and in nitrogen-rich conditions (+N). The graphs show the difference between the two conditions ([+N] − [−N]). Bottom panels: density of increases in Rad51 binding along the genome during meiosis in nitrogen-rich versus nitrogen depletion conditions. The locations of (1) sites of Rad51 binding present only in +N conditions and (2) sites for which +N / −N ≥ 2 for the peak values were identified and combined. Consistent with the lower origin efficiencies across the genome in −N (Figures 1A and 1B), we found very few Rad51 binding sites that are unique to or increased in −N compared to +N (Figure S2C). We therefore focused only on the enrichment in Rad51 binding in +N conditions. The graphs represent the numbers of these sites determined over continuous ∼200 kb windows, as in the top panel. In both the top and bottom panels, the abscissa represents the moving average of the chromosome coordinates for the corresponding ∼200 kb windows. Black triangles indicate a correspondence of the major peaks between the origin efficiency and Rad51 results, and white triangles denote differences. The differences in Rad51 binding between −N and +N conditions are reflected by differences in double-strand break formation, as assayed by pulsed-field gel electrophoresis (Figure S3C). (B) Increases in Rad51 binding in nitrogen-rich medium are clustered in areas with higher origin efficiencies in +N versus −N conditions. Changes in origin efficiencies as in (A) are in red. Circles indicate discrete sites of new or increased (≥2-fold) Rad51 binding in +N conditions (as determined in A). Representative genomic regions are displayed in greater detail. The x axis scale for the graph representing a region of chromosome 3 differs from those for chromosomes 1 and 2 for ease of visualization.

Taking advantage of this approach, we ascertained whether alterations in the pattern of origin selection affect the major steps of meiosis. We observed no apparent changes in the timing and execution of meiosis I and II relative to the completion of DNA replication (Figures 1F and 1H), and spore viability was unaffected (Figure 1I). However, the increased number of efficient origins in cells that undergo meiosis in continuous nitrogen-rich conditions (Figure S1C) suggested that the time required for genome duplication may be correspondingly altered. Using two complementary methods, flow cytometry and single-cell labeling of DNA synthesis (Supplemental Experimental Procedures), we found that the duration of premeiotic DNA replication in nitrogen-rich medium is around 25 min, compared to 50 min in the standard nitrogen-depleted conditions (Figures 2G and S1D). We conclude that in contrast to previous proposals (Holm, 1977, Cha et al., 2000), but consistent with the findings described by Blitzblau et al. (2012) in *S. cerevisiae*, overall meiotic progression does not require either the activation of a specific set of origins or a particular length of S phase.

### Origin Selection Determines the Sites of Double-Strand Break Formation

In many organisms, including fission yeast, the duration of premeiotic S phase has been characterized to be around twice that of S phase in mitotically cycling cells (Cha et al., 2000, Jaramillo-Lambert et al., 2007). This property was suggested to be critical for meiosis-specific events in DNA metabolism, in particular the subsequent recombination between homologous chromosomes (Holm, 1977, Cha et al., 2000), which is required for proper chromosome segregation and for generating diversity in the haploid products of meiosis (Roeder, 1997). In budding yeast meiosis, local changes in the timing of DNA synthesis led to corresponding changes in the timing of double-strand break (DSB) formation, which is required for recombination; however, the positions and frequencies of DSBs remained unaffected (Borde et al., 2000). Our results on premeiotic origin usage and S phase duration therefore prompted us to investigate the role of the program of DNA replication in the establishment of meiotic recombination, evaluating the impact of changing origin selection on this process.

We first determined the distribution of potential sites of recombination across the genome using the binding of the recombination protein Rad51/Rhp51 to DNA as a marker for DSBs (Roeder, 1997). Chromatin immunoprecipitation (ChIP) of Rad51 followed by microarray analysis (ChIP-on-chip) was performed for meiosis either after nitrogen starvation (−N) or in continuous nitrogen-rich conditions (+N). Rad51 localization in −N reflected the distribution of the sites of DSB formation (previously determined in the same conditions by assessing the sites of linkage of the meiotic transesterase Rec12 (Cromie et al., 2007; Figure S2A), validating our approach. In our experiments, we detected distinct peaks of Rad51 across the genome in both nutritional conditions and found unexpected increases in the number of Rad51 binding sites as well as in the level of binding in +N compared to −N (Figures S2B–S2D; see Figure S2E for quantitative PCR experiments at specific loci, confirming the genome-wide data). This indicates that the establishment of recombination occurs despite a reduction by half of the length of S phase and that DSBs are in fact formed in +N to an even greater extent than those in the conventional nitrogen-depleted meiotic conditions (see also Figure S3C). Given these observations, we then compared the changes in origin efficiencies and in Rad51 binding across the genome in −N versus +N conditions. Importantly, we found that between the two programs, the local differences in Rad51 binding closely correspond with local differences in origin efficiencies (Figures 2A and S3A). Our results reveal a clustering of the sites of new and increased Rad51 binding in regions with significantly increased origin usage during premeiotic S phase in +N as compared to −N (Figure 2B). These differences in Rad51 recruitment are not accompanied by alterations in chromatin features previously linked to sites of meiotic recombination, such as nucleosome occupancy and histone H3 lysine 4 trimethylation (Figure S3B). This indicates that the influence of origin activity on the subsequent recombination events is unlikely to be mediated by these features. The striking observation that changes in origin efficiencies between replication programs are associated with changes in Rad51 binding and DSB formation suggests that local increases in replication initiation events lead to preferential recruitment of recombination factors in these regions.

**Figure 3:**
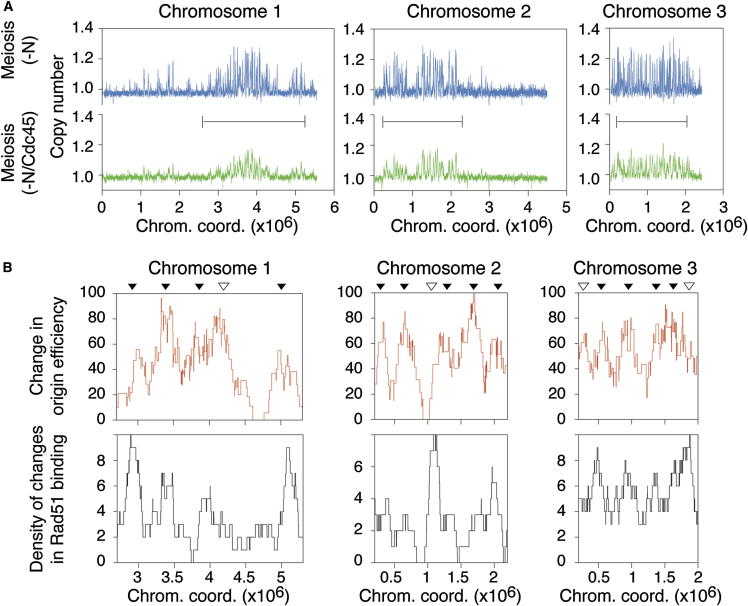
Modulation of Origin Efficiencies by Altering Levels of the Replication Machinery Induces Specific Changes in Rad51 Binding (A) Decrease in origin efficiencies across the genome as a result of the dominant-negative effect of *cdc45* overexpression in nitrogen starvation conditions. Replication profiles in strains induced to undergo meiosis after nitrogen depletion (−N) or after nitrogen depletion with altered Cdc45 levels (−N/Cdc45; see Experimental Procedures). The lines in each of the three graphs delineate the regions analyzed in Figure 3B. (B) Genome-wide analysis of changes in origin efficiencies and Rad51 binding in −N versus −N/Cdc45 conditions. As there are large areas of the genome with few or no apparent active origins above our detection threshold in both the −N and −N/Cdc45 conditions, only regions where there are sufficient origin densities and efficiencies for reliable analysis were considered (Figure S4A). Top panels: changes in origin efficiencies over continuous ∼200 kb windows (analysis as performed for Figure 2A). The difference between the two conditions is shown ([−N] − [−N/Cdc45]). Bottom panels: density of increases in Rad51 binding along the genome in −N compared to −N/Cdc45 conditions (analysis as in Figure 2A). The locations of (1) sites of Rad51 binding present only in −N conditions and (2) sites for which (−N) / (−N/Cdc45) ≥ 2 for the peak values were identified and combined over ∼200 kb windows. Consistent with the lower origin efficiencies across the genome in −N/Cdc45, we found very few Rad51 binding sites that are unique to or increased in −N/Cdc45 compared to −N (Figure S4B). In both the top and bottom panels, the abscissa represents the moving average of the chromosome coordinates for the corresponding ∼200 kb windows. Black triangles indicate a correspondence of the major peaks between the origin efficiency and Rad51 binding results, and white triangles denote differences.

Next, to further demonstrate the coupling of origin activity with meiotic recombination, we altered origin usage by directly affecting the replication machinery via modulating the levels of the preinitiation complex component Cdc45 without changing nutritional state. Decreasing *cdc45* expression resulted in a significantly delayed entry into S phase and a defect in S phase completion, preventing analysis and comparison of the subsequent meiotic recombination patterns (data not shown). However, we reasoned that in −N conditions, where the levels of critical replication factors including Mcm proteins and Cdc18 are significantly reduced (Namdar and Kearsey, 2006), overexpression of *cdc45* is likely to result in a dominant-negative effect via the titration of key components in the machinery, thereby reducing origin efficiencies. In the nitrogen depletion conditions described above, strong overexpression of *cdc45* (−N/Cdc45) (data not shown) led to a genome-wide decrease in origin efficiencies as compared to nitrogen depletion alone (Figures 3A and S4A). This allowed us to analyze the changes in both origin activity and Rad51 binding between these two conditions (−N versus −N/Cdc45). First, we observed an overall reduction in Rad51 binding across the genome in −N/Cdc45 that paralleled the reduction in origin usage in these conditions (Figure S4B; see also Figure S3C for DSB formation). We then assessed whether local changes in origin efficiencies were accompanied by local changes in Rad51 binding. The replication programs in both −N and −N/Cdc45 show regions of the genome in which few or no apparent active origins above our threshold can be detected (Figure S4A). Therefore, we focused our analysis on areas in which origin densities and efficiencies can be reliably determined. Our results demonstrate that there is an enrichment of sites of Rad51 binding that are increased or only present in −N compared to −N/Cdc45 in regions with correspondingly higher origin usage (Figure 3B). As Cdc45 is a key regulator of origin activation (Wu and Nurse, 2009), these findings provide evidence for a direct link between origin firing and meiotic recombination.

### Changes in Origin Activity Lead to Changes in Meiotic Recombination

**Figure 4:**
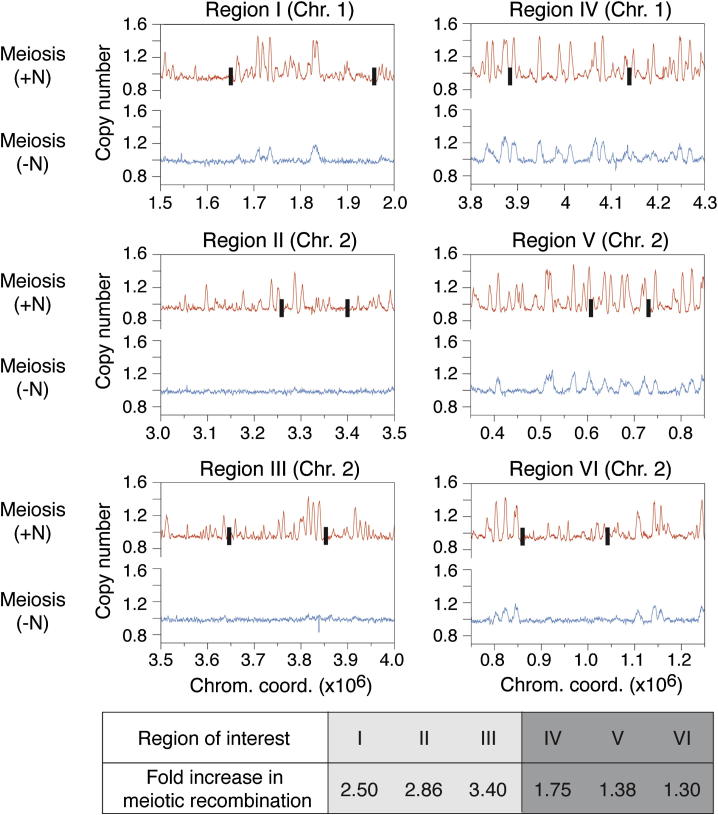
Changes in Origin Efficiencies Result in Changes in Meiotic Recombination Frequencies Meiotic recombination frequencies were determined after nitrogen depletion and in continuous nitrogen-rich conditions by random spore analysis (Experimental Procedures). Six positions were analyzed based on regions that show either strong (I, II, III) or limited (IV, V, VI) differences in origin efficiencies between programs (see Figure S3D for selection of the regions of interest), taking into account differences in both peak amplitudes and numbers. Blue, origin usage in meiosis after nitrogen depletion; red, origin usage in meiosis in continuous nitrogen-rich conditions. The sites of integration of antibiotic resistance cassettes used as markers in the recombination assays are indicated by the black boxes. The table shows the ratios of recombination frequencies in continuous nitrogen-rich medium compared to those after nitrogen depletion (+N/−N).

Given these results, we tested whether the changes in DSB formation due to modulation of the replication program affect the outcome of meiotic recombination by determining recombination frequencies at a number of sites across the genome. To this end, we used a series of diploid strains containing heterozygous markers in regions that show either strong or minimal differences in origin efficiencies between the −N and +N conditions (Figures 4 and S3D). We assayed the number of recombinant spores produced when cells undergo meiosis after nitrogen starvation or in continuous nitrogen-rich conditions and evaluated the fold change in recombination frequencies at the marked loci (+N/−N). Our data show that the extent of the changes in origin usage between programs, which correlate with those in Rad51 binding, is reflected by differences in recombination frequencies (Figure 4). Taken together, these results implicate origin selection as an important determinant in the establishment of the sites of meiotic recombination.

## Discussion

Our studies have identified the program of DNA replication as a critical component in the organization of meiotic recombination. We demonstrate that although a specific pattern of origin usage is neither induced by meiotic commitment nor required for the major steps of meiotic progression, origin efficiency significantly affects recombination factor recruitment and recombination frequencies. While previous studies have found that duplication of the entire genome is not required for DSB formation (Blitzblau et al., 2012, Murakami and Nurse, 2001), we have specifically shown that origin selection regulates the distribution of the sites of meiotic recombination. Setting up recombination likely depends on additional layers of control, such as chromatin environment and chromosomal context, thereby limiting the correlation between origin activity and recombination within a given program. However, in our experimental system, generating major changes in origin usage during premeiotic S phase and comparing identical regions of the genome in different conditions has allowed us to link the organization of genome duplication with the exchange of genetic material during meiosis.

How might the initiation of DNA synthesis promote meiotic recombination? Our results demonstrate that decreasing origin efficiencies through changes in Cdc45 levels leads to a reduction in Rad51 binding, suggesting that components of the replication machinery may be directly involved. One possible connection may be through the conserved cyclin-dependent protein kinase (CDK) and Dbf4-dependent kinase (DDK), which are required for origin activity as well as for recruiting the DSB protein Spo11 in budding yeast (Rec12 in fission yeast). Preferential localization of these kinases in regions of active origins could phosphorylate the Mer2/Rec15 protein, a target of both kinases (Sasanuma et al., 2008, Wan et al., 2008). As these steps are critical for loading Spo11/Rec12 for DSB formation, this could then increase local recombination frequencies (Murakami and Keeney, 2008).

Our observations in fission yeast appear to differ from those previously made in budding yeast. In *S. cerevisiae*, deletion of origins in a region leads to a local delay in replication and a corresponding delay in the formation of DSBs (Borde et al., 2000). Consistent with this, changing origin usage and timing of replication in *S. pombe* changes the timing of DSB formation (Figures S2E and S3C). However, this also alters the frequencies of DSBs in the affected domain. So although budding yeast may maintain a more constant frequency of DSBs than fission yeast through a process of DSB homeostasis (Carballo et al., 2013), the existence of a link between origin selection and DSB formation seems to be common to both models and may be mediated by the same factors.

Coupling origin usage with recombination could serve as a mechanism to protect the genome in adverse growth conditions. Given the sensitivity of origin selection to the nutritional state, this may provide a means of limiting recombination in conditions in which there is a risk that it may not proceed to completion, as even one unrepaired DSB can result in lethality (Bennett et al., 1993). Conversely, in better growth conditions in which origin firing is increased, initiating higher levels of recombination may promote the exchange of genome complements, generating diversity for evolutionary change.

This work provides evidence for functional consequences of genome-wide changes in origin usage. Our results imply that origin selection can play an integral role in regulating cellular processes, suggesting that the reprogramming of DNA replication observed in developmental and pathological situations may contribute to the associated physiological states.

## Experimental Procedures

### Strains and Growth Conditions

Standard methods for fission yeast were used (Hayles and Nurse, 1992, Moreno et al., 1991). Strains used in this study are listed in Table S1. Experiments were performed in minimal medium plus supplements (EMM4S) unless otherwise indicated. Methods for synchronous meiotic induction in different conditions are described in Figure S1A.

To modulate Cdc45 levels, the *cdc45* promoter was replaced at its locus with the thiamine-repressible *nmt1* promoter. After nitrogen starvation, repression of *nmt1* by addition of thiamine reduces Cdc45 levels to such a degree that entry and completion of S phase are compromised. In contrast, in the absence of thiamine, *cdc45* is strongly overexpressed (data not shown), resulting in S phase prolongation linked to reduced origin usage across the genome (Figure 3). This likely occurs through a dominant-negative effect of high Cdc45 levels, which may titrate out other critical limiting components of the machinery in nitrogen starvation conditions, where there is large-scale protein degradation and a significant reduction in the level of replication factors such as Mcms and Cdc18 (Nakashima et al., 2006, Namdar and Kearsey, 2006).

### Recombination Assays

Strains for analysis of recombination frequencies were constructed by integration of antibiotic resistance markers in intergenic regions (Bähler et al., 1998) (positions indicated in Figures 4 and S3D). Meiosis in *pat1-114* diploids heterozygous for the markers was induced in −N or +N conditions (Figure S1A). Asci were harvested at least 10 hr (−N) and 8 hr (+N) after shift to restrictive temperature, and spores were plated in equal numbers on rich medium (YE4S). Colonies were genotyped by replica plating to plates containing G418 and hygromycin B or G418 and nourseothricin.

### Cell-Cycle Analysis

For flow cytometry, cells were fixed in 70% ethanol, treated with 0.1 mg/ml ribonuclease A (RNase A), stained with 2 μg/ml propidium iodide, and analyzed using a BD FACSCalibur. To assess meiotic progression, cells were heat fixed on microscope slides and stained with 2 μg/ml DAPI to visualize nuclei. For microscopy (Figure 1), live cells were stained with Hoechst (DNA) and Blankophor (cell wall).

### Single-Cell Analysis of S Phase Length

*pat1-114* diploids that incorporate thymidine analogs into newly synthesized DNA (Sivakumar et al., 2004) were induced to undergo synchronous meiosis in −N or +N conditions. Cells were labeled with EdU either at the time of the temperature shift (total labeling) or at specific time points (pulse labeling). Detection was performed using the Click-iT EdU Imaging Kit (Invitrogen). The description of the assessment of S phase length using this method is described in the Supplemental Experimental Procedures.

### Chromatin Immunoprecipitations and Microarrays

ChIP was performed as in Wu and Nurse (2009). For Rad51 binding, ChIP-on-chip samples were taken 1 hr after S phase completion in all conditions, at which time the level of Rad51 binding across the genome reaches its peak (after *pat1-114* inactivation, 4 hr [−N], 2 hr [+N], and 5 hr [−N/Cdc45]) (see also Figure S2E). For ChIP using histone H3 and histone H3 K4 trimethyl antibodies, samples were taken at the start of S phase in both conditions, when origin activation is established (2 hr and 30 min after *pat1-114* inactivation for −N and +N, respectively). Antibodies used included Rad51 (H-92, Santa Cruz; previously used in Caspari et al., 2002 to detect fission yeast Rad51), histone H3 (ab1791, Abcam), and histone H3 K4 trimethyl (ab8580, Abcam). For ChIP-on-chip experiments, ChIP material was amplified and labeled as in van Bakel et al. (2008). Each ChIP was compared to its reciprocally labeled input sample.

For origin efficiencies, genomic DNA was harvested prior to DNA synthesis and during S phase after treatment with HU. Genomic DNA samples were labeled using the BioPrime Plus Array CGH Indirect Genomic Labeling System (Invitrogen) and hybridized to microarrays; dye-swap experiments were performed to reduce noise.

All experiments used Agilent 4×44k *S. pombe* arrays (60-mer oligos, ∼250 nt resolution). Details for the analysis of microarray data are provided in the Supplemental Experimental Procedures.

## Accession Numbers

The array data reported in this paper have been deposited in the NCBI GEO database with the accession number GSE53921.

## Supplemental Information

Document S1. Supplemental Experimental Procedures, Figures S1–S4, and Table S1
