# Supplementary material for: Replication Origin Selection Regulates the Distribution of Meiotic Recombination
Source: Mol Cell. 2014 Feb 20;53(4):655–62. doi: 10.1016/j.molcel.2014.01.022 (PMC3988929; doi:10.1016/j.molcel.2014.01.022)
Supplement: Document S1. Supplemental Experimental Procedures, Figures S1–S4, and Table S1 [file mmc1.pdf]

Molecular Cell, Volume *53*

Supplemental Information

Replication Origin Selection Regulates the Distribution of Meiotic Recombination

Pei-Yun Jenny Wu and Paul Nurse

Figure S1

A

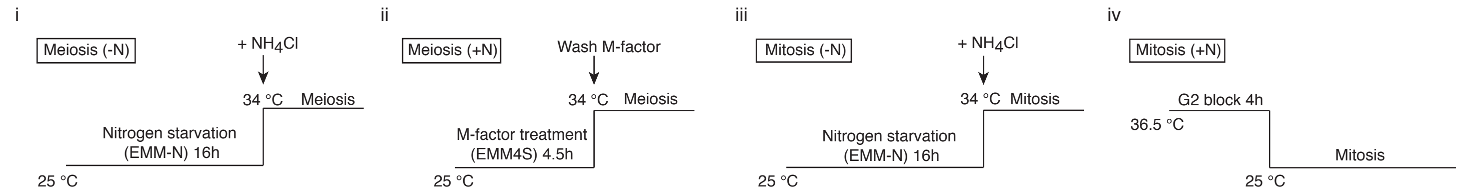

B

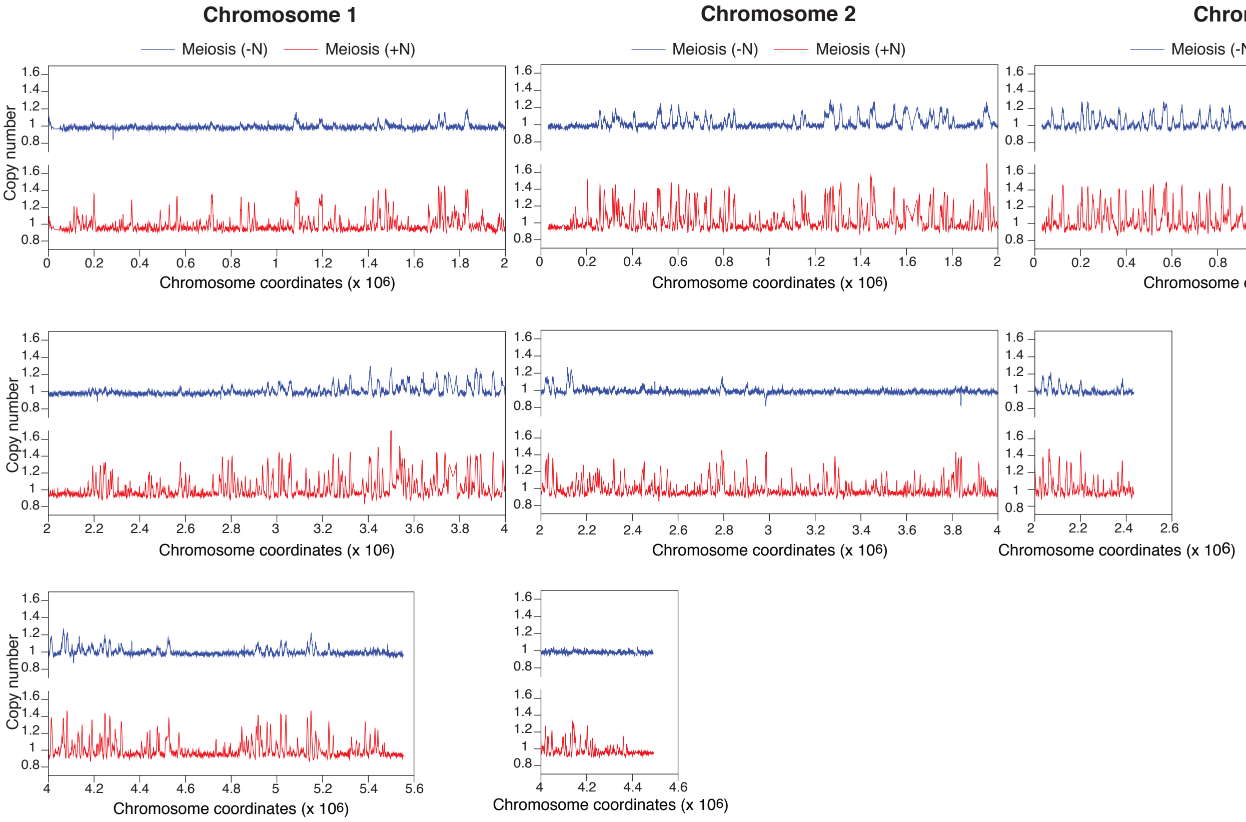

C

Origin usage in pre-meiotic S phase

	- N	+ N
**Chromosome 1**	60	210
**Chromosome 2**	64	181
**Chromosome 3**	66	125
**Total**	190	516

D

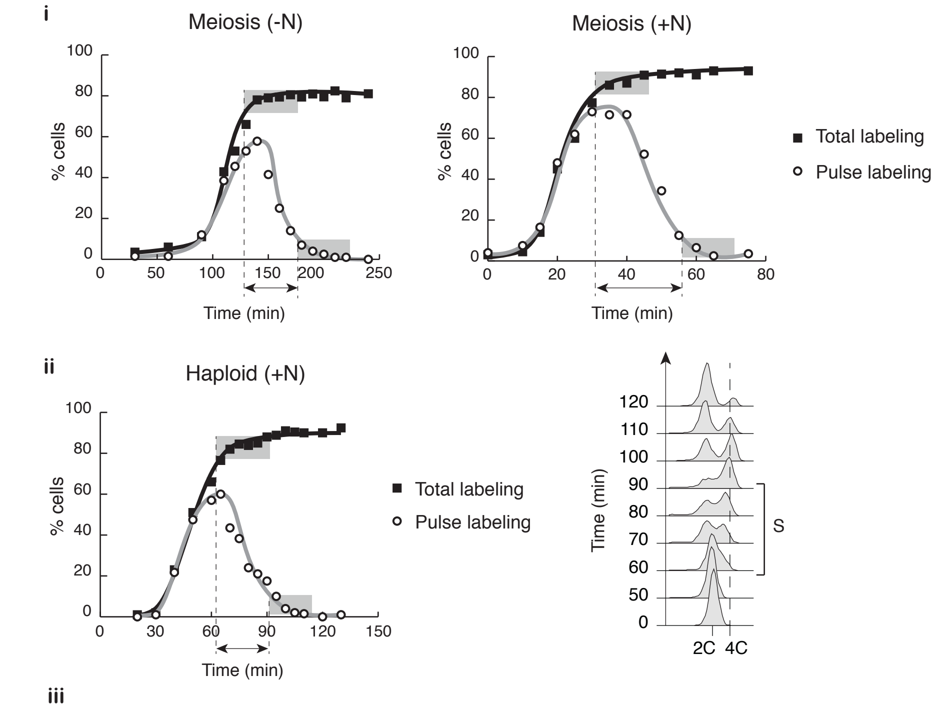

iii

	Meiosis (-N)	Meiosis (+N)	Haploid (+N)	Diploid (-N)
S phase length (min)	50	25	30	50

Figure S1 (related to Figure 1)

Program of DNA replication and S phase progression in -N and +N conditions

(A) Experimental design for initiating synchronous DNA replication in different conditions. **(i, ii)** Diagrams of methods for inducing meiosis after nitrogen depletion (-N) and in continuous nitrogen-rich (+N) conditions. To determine origin efficiencies, cells were treated with 12 mM HU at the time of shift to restrictive temperature. **(i)** Meiosis after nitrogen depletion. Diploid *pat1-114 h+* cells were nitrogen-starved in minimal medium (EMM-N) for 16 h at 25 ºC, re-fed nitrogen (0.05% w/v NH_4Cl), and shifted to 34 ºC to induce meiosis. Cells were harvested after the end of S phase (4 h after the shift) for array experiments. **(ii)** Meiosis in continuous nitrogen-rich conditions. Diploid *pat1-114 h+* cells were grown in supplemented minimal medium (EMM4S) at 25 ºC and then treated with M-factor (Dr. Brian Chait, Rockefeller University) for 4.5 h to induce a G1 arrest. At this time, M-factor was removed by filtration and cells were shifted to 34 ºC to induce synchronous meiosis. Cells were harvested after the end of S phase (1 h after the shift) for array analysis. **(iii, iv)** Diagrams of methods for determining origin efficiencies in vegetative cells after nitrogen starvation (-N) or in nitrogen-rich conditions (+N). Cells were treated with 12 mM HU at the start of the synchronous mitotic cycle. **(iii)** Haploid or diploid wild type cells were nitrogen-starved for 16 h at 25 ºC, then re-fed nitrogen (0.05% w/v NH_4Cl) for re-entry into the mitotic cycle and shifted to 34 ºC (same conditions as in **i** but in the absence of the *pat1-114* mutation). Cells were harvested 4 h after the shift to assess origin usage. **(iv)** Exponentially growing haploid *cdc25-22* cells grown in EMM4S were shifted to 36.5 ºC for 4 h to arrest cells in G2, then shifted down to 25 ºC for synchronous mitotic cycles. For array experiments, cells were harvested 90 min after the return to permissive temperature.

(B) Pre-meiotic replication programs in -N and +N conditions. Genome-wide representation of origin efficiencies in pre-meiotic S phase as determined by microarray analysis of copy number in two conditions: after nitrogen starvation (-N) and in continuous nitrogen-rich conditions (+N) (the graphs represent a detailed view of the data in Fig. 1). The geometric mean of continuous windows of five probes was determined for each dataset before averaging dye-swap experiments (see Supplemental Experimental Procedures). The abscissa represents the moving average of the chromosome coordinates for the corresponding groups of probes.

(C) Origin usage in pre-meiotic S phase. Changes in the number of origins (1.1 cutoff for copy number analysis by microarrays, similar to (Heichinger et al., 2006)) used during pre-meiotic S phase after nitrogen depletion (-N) and in nitrogen-rich medium (+N).

(D) Determination of the length of pre-meiotic S phase. Single-cell analysis of S phase length using fluorescence detection of incorporated thymidine analogues (see Supplemental Experimental Procedures). To determine the duration of S phase, we assumed that each cell in a population takes the same amount of time to attain bulk genome duplication. Making this assumption, we performed labeling experiments in cells undergoing synchronous S phase during mitosis or meiosis (see Fig. S1A) to determine at any given time both 1) the total percentage of cells that have already initiated S phase (black squares and lines in **i** and **ii**, total labeling) and 2) the percentage of cells still in S phase (open circles and gray lines in **i** and **ii**, pulse labeling). The last 10% of cells that enter S phase (gray box on the black line) will also be the last cells to exit S phase (gray box on the gray line). Therefore, the time between S phase entry and exit of this population represents an estimate of the length of S phase (indicated by double-headed arrows). **(i)** The length of pre-meiotic S phase after nitrogen-starvation is around 50 min (Meiosis (-N)), while it is only around 25 min for nitrogen-rich medium (Meiosis (+N)). T=0 corresponds to the time of shift of *pat1-114* diploids to restrictive temperature. **(ii)** As a reference, the duration of mitotic S phase in cells synchronized using *cdc25-22* (Haploid (+N)) was determined by this method to be 30 min (left panel), consistent

with our flow cytometry analysis (right panel) and known estimates of S phase length in these conditions. T=0 corresponds to the time of shift to permissive temperature. **(iii)** Table summarizing the lengths of S phase in different conditions. Diploid (-N) is a control for S phase length in wild type diploid cells after nitrogen-starvation (raw data not shown) treated as in Fig. S1A(iii).

Figure S2

A

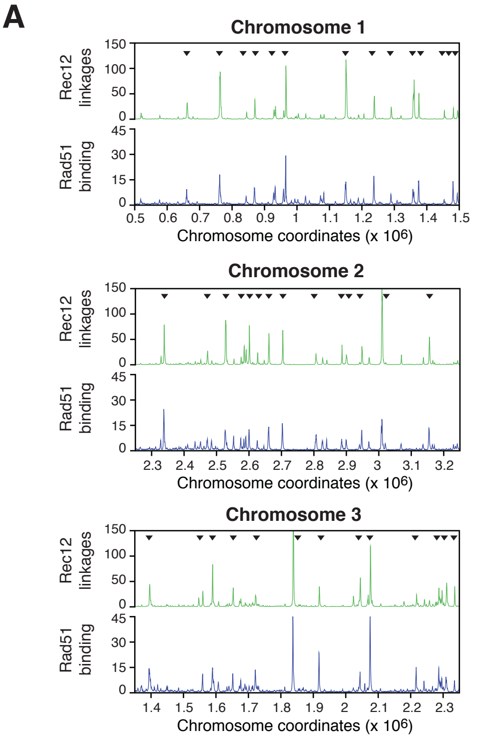

D

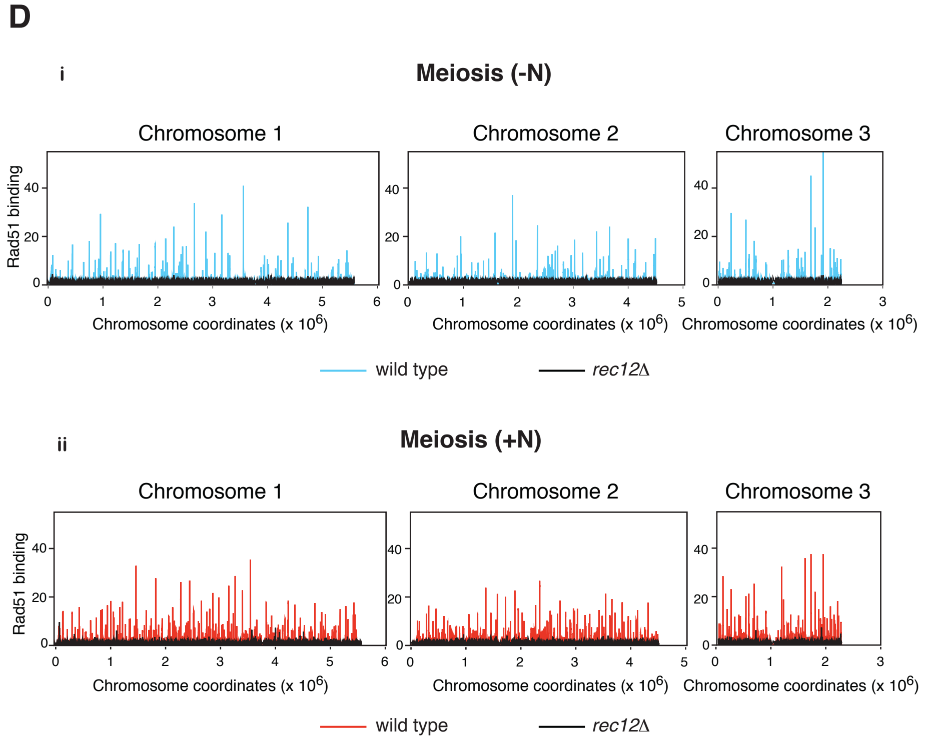

B

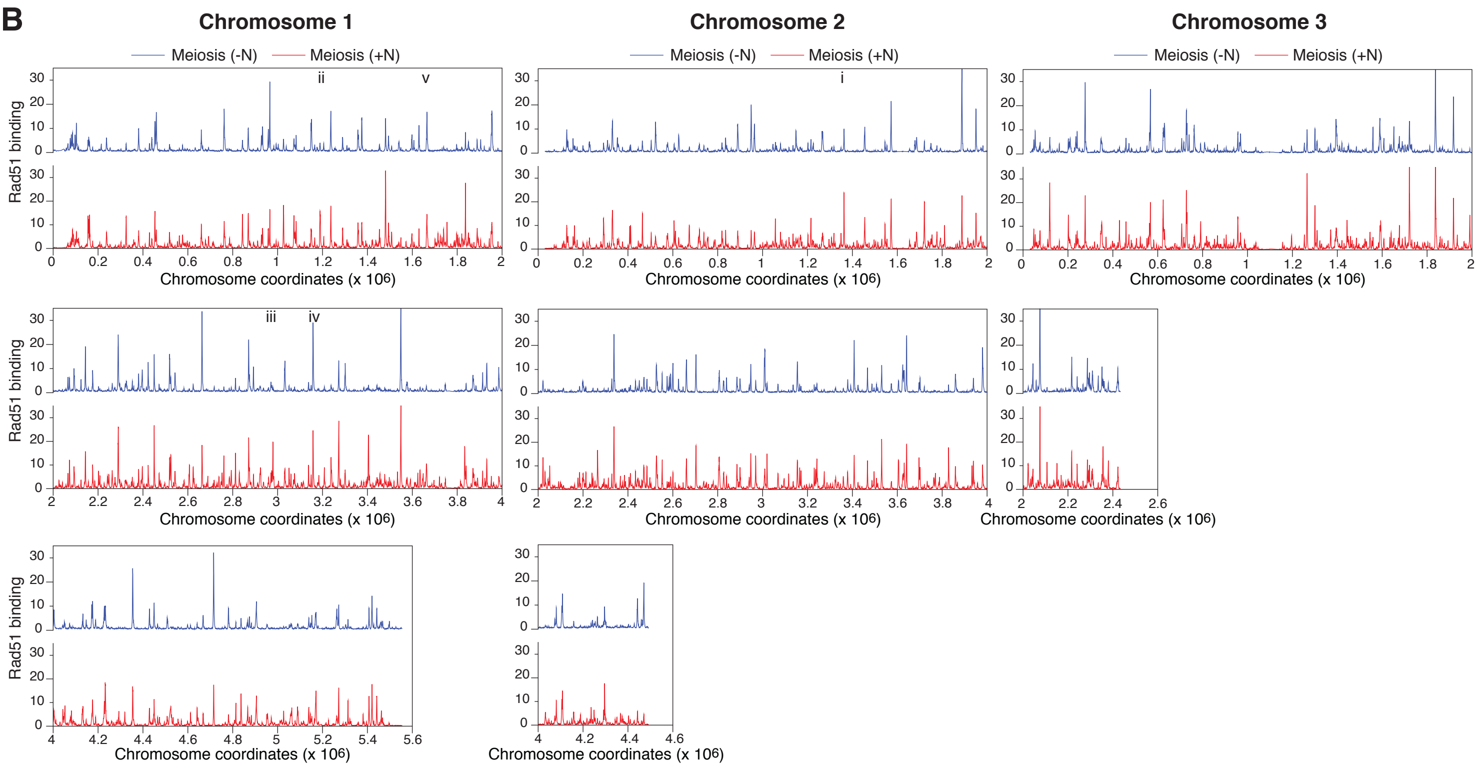

C

Sites of increased Rad51 binding during meiosis

	Nitrogen-rich (+N)			Nitrogen-depleted (-N)		
	Unique	Increased	Total	Unique	Increased	Total
Chromosome 1	199	75	430	19	3	250
Chromosome 2	167	73	359	5	8	196
Chromosome 3	81	41	218	12	3	149
Total	447	189	1007	36	14	595

E

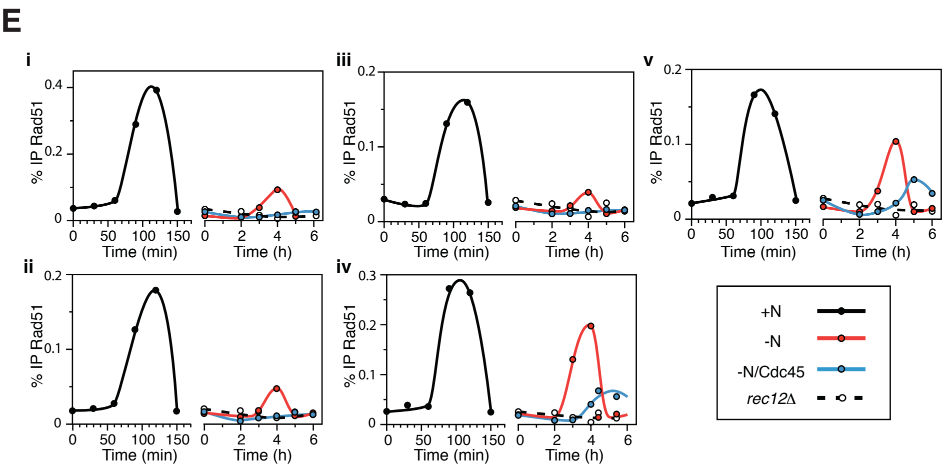

Figure S2 (related to Figures 2 and 3)

Rad51 binding in -N and +N conditions

(A) Overlap between the genome-wide DNA binding of Rad51 and Rec12 linkages. The distribution of Rad51 binding sites in meiosis after nitrogen-starvation (blue) as determined by our ChIP-on-chip analysis (see Experimental Procedures) is highly reflective of previously published data for Rec12 linkages at DSBs (green) (Cromie et al., 2007; Hyppa et al., 2008). Both experiments were performed in *pat1-114* diploid cells that were first nitrogen-starved, then re-fed nitrogen and induced to undergo meiosis by shift to the restrictive temperature. Samples were collected to assess Rad51 binding 4 h after the shift to restrictive temperature, one hour after the completion of S phase (see Fig. S1A). The data for Rec12 are taken from the 5 h time point in Dataset S2 of Hyppa et al., 2008. The same arrays were used as in our experiments, and we have treated the raw data in the same way for both the Rec12 and Rad51 datasets for direct comparison. The geometric means of continuous windows of five probes are shown on the graphs for Rec12. For Rad51 binding, the 5-probe geometric means of our two datasets were averaged. Representative regions for each of the three fission yeast chromosomes are shown. The abscissa represents the moving average of the chromosome coordinates for the corresponding groups of probes, and the ordinate shows the levels of Rad51 binding and Rec12 linkages. The positions of major peaks shared between Rec12 and Rad51 are marked by triangles. The Pearson correlation coefficients for Rec12 linkages and Rad51 binding were calculated for each chromosome: (**1**) 0.683; (**2**) 0.676; (**3**) 0.561; these indicate strong positive correlations between the datasets.

(B) Rad51 binding to DNA in -N and +N meiosis. Genome-wide representation of Rad51 binding during meiosis after nitrogen-depletion (-N, blue) or in continuous nitrogen-rich conditions (+N, red). A greater number of peaks as well as an increase in peak heights were observed in +N conditions (see Fig. S2C for quantification). Each dataset represents the average of two experiments; for each experiment, the geometric mean of continuous windows of five probes was first determined before averaging. The abscissa represents the moving average of the chromosome coordinates for the corresponding groups of probes, and the ordinate shows the level of Rad51 binding. The binding of Rad51 in both -N and +N conditions is specific to DSB formation during meiotic recombination, as this requires the Rec12 transesterase (see Fig. S2D). The positions indicated by Roman numerals i-v refer to the loci assessed in Fig. S2E for Rad51 binding in time course experiments.

(C) Sites of increased Rad51 binding during meiosis. The table shows the numbers of identified Rad51 peaks in meiosis after nitrogen starvation (-N) or in nitrogen-rich conditions (+N). Peaks which are only found in nitrogen-rich conditions (+N, Unique) or which show a two-fold or greater increase over nitrogen-depletion (+N, Increased) were identified. The same assessment was performed for sites found only after nitrogen depletion (-N, Unique) or which show a two-fold or more change compared to nitrogen-rich conditions (-N, Increased). Consistent with the lower origin efficiencies across the genome in -N, (Fig. 1A-B), we found very few Rad51 binding sites that are unique to or increased in -N conditions. For our subsequent analyses (Fig. 2), we therefore focused only on the enrichment in Rad51 binding in +N conditions.

(D) Rad51 binding to DNA in -N and +N meiosis requires Rec12. Genome-wide representation of Rad51 binding during meiosis after nitrogen-depletion (-N, top panel) or in continuous nitrogen-rich conditions (+N, bottom panel). **(i)** ChIP-on-chip analysis of Rad51 in wild-type (blue line) and *rec12Δ* (black line) strains in the -N condition. **(ii)** ChIP-on-chip analysis of Rad51 binding in wild type (red line) and *rec12Δ* (black line) strains in the +N condition. For all panels, the geometric mean of continuous windows of five probes was determined for each dataset. The abscissa represents the moving average of the chromosome coordinates for the corresponding groups of

probes, and the ordinate shows the level of Rad51 binding. In both the -N and +N conditions, there is virtually no detectable Rad51 binding in *rec12Δ* cells.

(E) Time course of Rad51 binding. ChIP analysis of Rad51 binding at five distinct loci in different strains and conditions. All experiments were performed using *pat1-114* diploid cells carrying the indicated genotypes and treated in the same conditions as for the ChIP-on-chip experiments. +N: nitrogen-rich conditions (black circles, black line); -N: nitrogen depletion (red circles, red line); -N/Cdc45: nitrogen depletion of cells with altered levels of Cdc45, leading to reduced origin efficiencies across the genome (see Fig. 3 and Fig. S4; blue circles, blue line); *rec12*Δ: nitrogen depletion of mutants deleted for *rec12* (open circles, dashed line). T=0 represents the time of the shift to restrictive temperature. The time points used for the ChIP-on-chip experiments correspond to those of the peaks of Rad51 binding in +N, -N, and -N/Cdc45 in these assays. The relative levels of Rad51 binding at these loci are consistent with what we observe in our genome-wide assays. The chromosomal positions of each locus are as follows: i) chromosome 2, 1.362 x 10^6; ii) chromosome 1, 1.189x10^6; iii) chromosome 1, 2.979 x 10^6; iv) chromosome 1, 3.158x10^6; v) chromosome 1, 1.665 x 10^6. These positions are shown in Figure S2B.

Figure S3

A

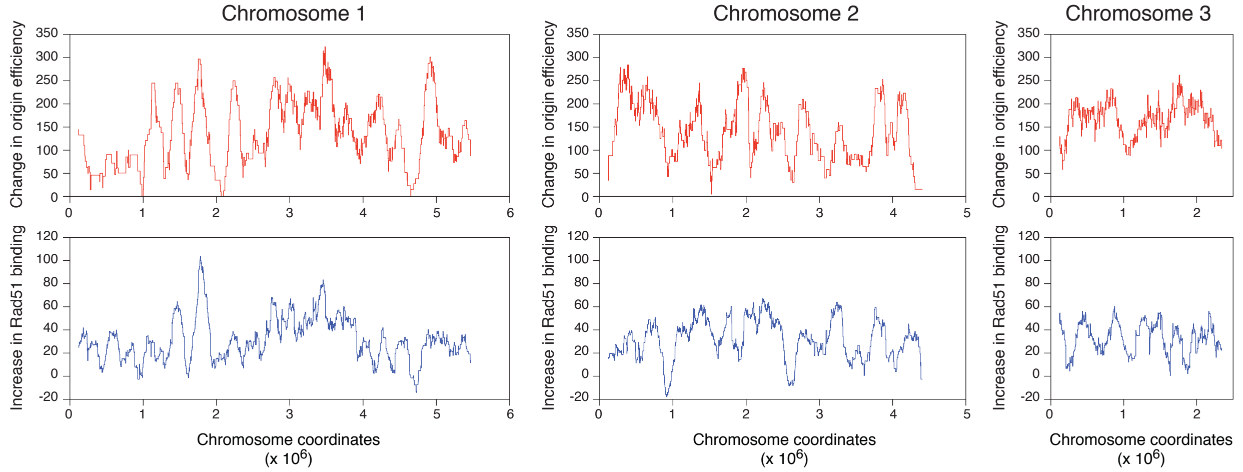

B

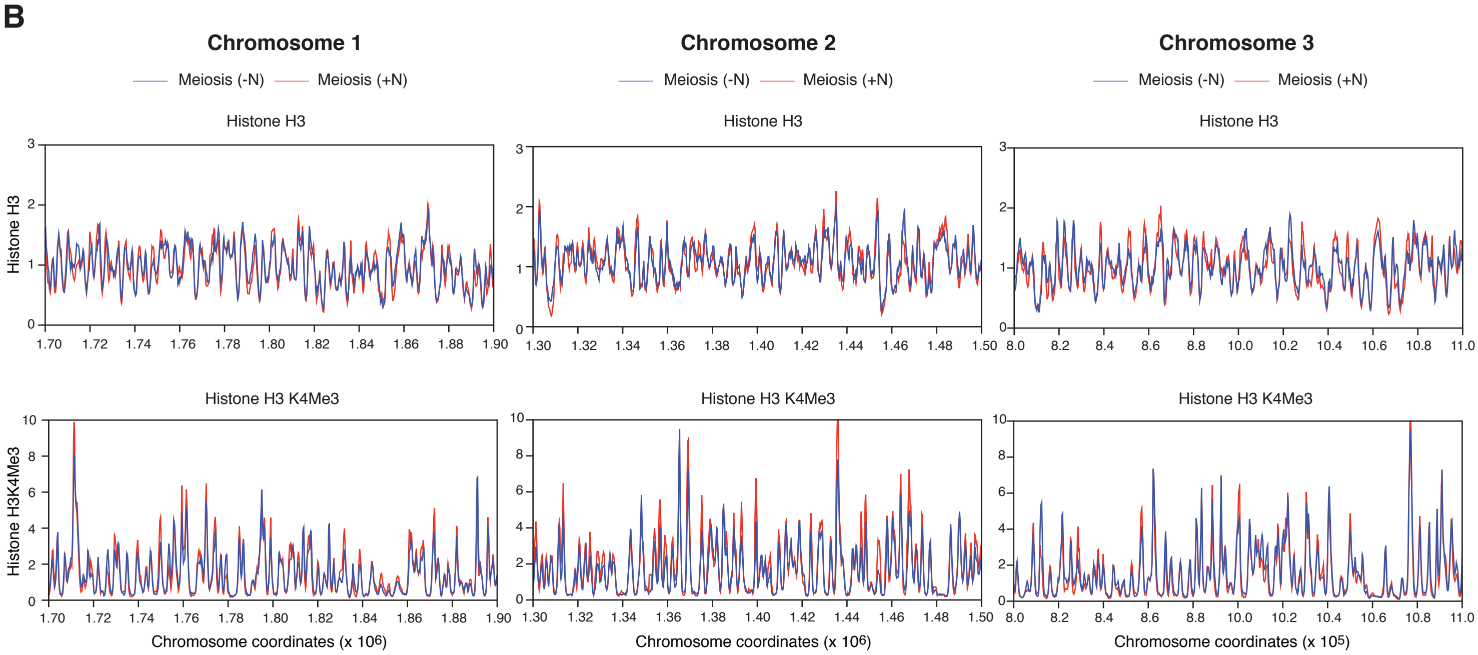

C

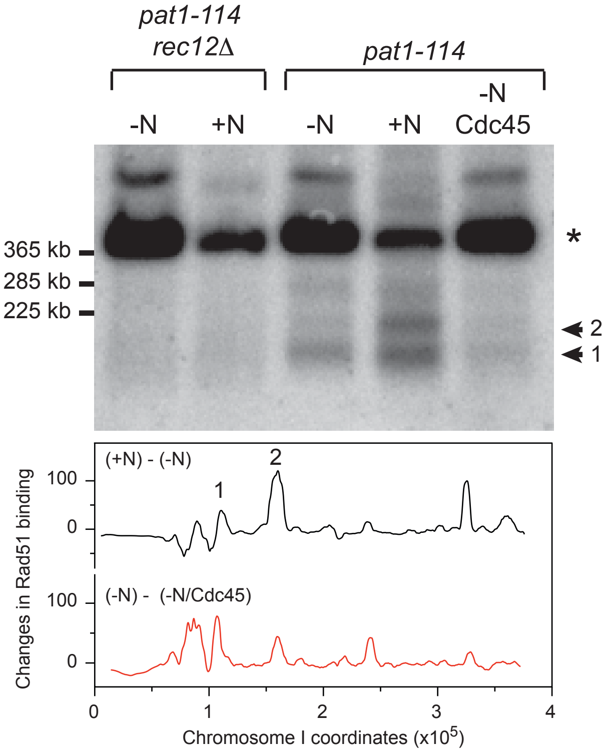

D

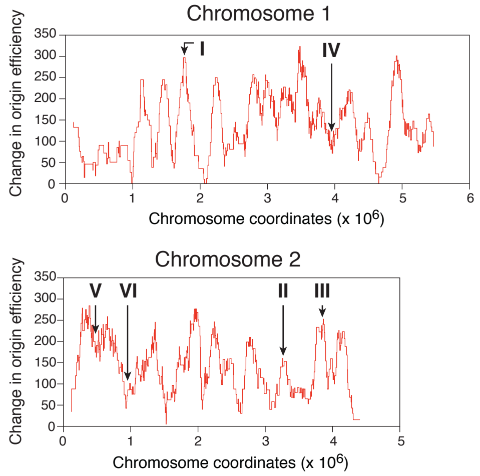

Region	+N recombinant	+N total	-N recombinant	-N total
I	177	2310	80	2494
II	170	9280	61	9532
III	143	1838	32	1401
IV	444	4064	245	3930
V	300	9346	174	7484
VI	292	4645	177	4054

Figure S3 (related to Figures 2, 3, and 4)

Changes in Rad51 binding and in recombination frequencies between different replication programs reflect changes in origin efficiencies

(A) Correlation between changes in origin efficiencies and the level of Rad51 binding. In contrast to Fig. 2A, which looks at the density of changes in Rad51 binding, this figure directly compares the difference in the levels of Rad51 binding (taking into account peak heights and positions) with changes in origin efficiencies. Top panels: Alterations in origin efficiencies in pre-meiotic S phase as shown in Fig. 2. The sums of origin efficiencies were determined for continuous ~200 kb windows for pre-meiotic S phase after nitrogen depletion (-N) and in nitrogen-rich conditions (+N). The difference between the two conditions is shown [(+N) - (-N)]. Bottom panels: Increases in Rad51 binding along the genome during meiosis in +N as compared to -N. For each condition, peaks of Rad51 binding were identified, and their values were added over continuous ~200 kb windows. The graph represents the subtraction of the values for nitrogen depletion from those for nitrogen-rich medium [(+N) - (-N)]. The abscissa represents the moving average of the chromosome coordinates for the corresponding ~200 kb windows. These data are complementary and consistent to those in the bottom panel of Fig. 2A.

(B) Differences in Rad51 binding in -N and +N meiosis are not correlated with chromatin features associated with meiotic recombination. ChIP-on-chip experiments showing the distribution of histone H3 (top panel) and histone H3 K4 trimethylation (bottom panel) during meiosis after nitrogen-depletion (-N, blue) or in continuous nitrogen-rich conditions (+N, red). The geometric mean of continuous windows of five probes was determined for each dataset. The abscissa represents the moving average of the chromosome coordinates for the corresponding groups of probes, the ordinate shows the levels of H3 or H3 K4 trimethylation. Representative regions of the genome are shown. Despite the minimal differences between the two conditions for both H3 and H3 K4 trimethylation, the datasets were subjected to the same analysis as that performed for Rad51 binding, and we observed no correlations between the changes in Rad51 binding in -N vs. +N and the changes in either histone occupancies or H3 K4 trimethylation (data not shown). Differences found between the -N and +N conditions for these features were in the same range as those observed between biological replicates, suggesting that these were within the noise of the experiments. Our results are consistent with those recently reported in budding yeast, which indicate that H3 K4Me3 has limited predictive power for determining the location and frequency of DSBs (Tischfield and Keeney, 2012).

(C) Increased Rad51 binding is associated with increased double-strand break formation. Top panel: DSB formation during meiosis after undergoing different programs of pre-meiotic S phase. High molecular weight genomic DNA was isolated from cultures of diploid *pat1-114* strains and digested using the NotI restriction enzyme. DNA was then separated by pulsed-field gel electrophoresis and analyzed by Southern blot using a probe for the left end of NotI fragment L on chromosome 1. This fragment shows changes in Rad51 binding between different strains and conditions (bottom panel). The following strains and conditions were analyzed: *pat1-114 rec12*Δ in nitrogen-rich (+N) and nitrogen depletion (-N) conditions; *pat1-114* in nitrogen-rich (+N) and nitrogen depletion (–N) conditions; *pat1-114* cells with altered levels of Cdc45 that show reduced origin efficiencies, nitrogen depletion (-N/Cdc45). Approximate molecular weights based on ethidium bromide staining are indicated (data not shown). The asterisk indicates full-length NotI fragment L, and lower molecular-weight bands (1 and 2) represent the results of DSB formation in this fragment. The relative abundance of these smaller fragments between strains and conditions is consistent with our genome-wide Rad51 binding data. (Bottom panel) Changes in Rad51 binding in NotI fragment L between different conditions. Taking an approach similar to that of Fig. S3A, data from ChIP-on-

chip analyses of Rad51 binding within this NotI fragment were added over continuous ~20 kb windows for each condition, and the graphs represent: (+N) – (-N): subtraction of the values for nitrogen depletion from those for nitrogen-rich conditions; (-N) – (-N/Cdc45): subtraction of the values for nitrogen-depletion in strains with altered Cdc45 (see Fig. 3 and Fig. S4) from those for nitrogen depletion. The raw data for Rad51 binding are shown in Fig. S2B and Fig. S4B. The abscissa represents the moving average of the chromosome coordinates for the corresponding ~20 kb windows.

(D) Raw data for the comparisons of recombination frequencies shown in Fig. 4. Six different sets of markers were analyzed (locations indicated in the diagram). Note that changes in origin efficiencies are shown over ~200 kb windows and take into account differences in both peak amplitudes and numbers in the regions of interest (see Fig. 4), providing a view of the overall differences between the two conditions. Regions I-III ("peaks") show strong differences, Regions IV-VI ("troughs") show limited differences. Note that the relatively high change in efficiency of the Region V "trough" is due to the high differences in origin efficiencies in the surrounding areas, which influence the values within the ~200 kb windows. Markers were not selected on chromosome 3 as the differences between peaks and troughs is less pronounced. Meiosis in *pat1-114* diploids heterozygous for different sets of markers (one antibiotic resistance cassette on each homologue) was induced after nitrogen-starvation (-N) or in nitrogen-rich medium (+N) as in Fig. S1A. Spores were isolated and tested for crossovers in the indicated intervals using the resistance cassettes. The first and third columns show the number of crossover events detected (recombination generating spores with two antibiotic resistance markers), and the second and fourth columns display the total number of spores scored. These results show that the differences in Rad51 binding between replication programs represent differences not only in the initiation but also the execution of recombination.

Figure S4

A

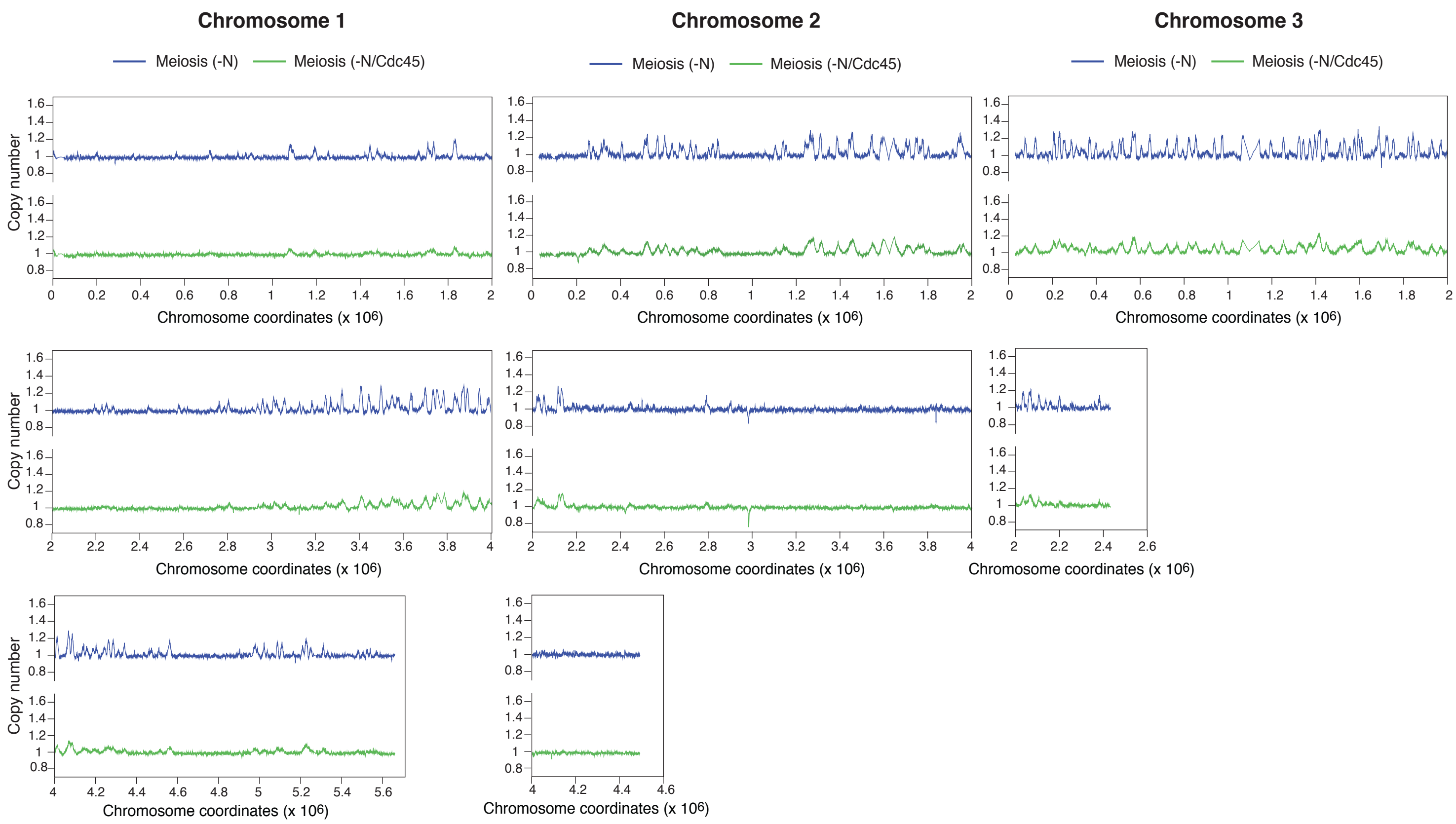

Chromosome 1
Chromosome 2
Chromosome 3
Meiosis (-N)
Meiosis (-N/Cdc45)
Copy number
Chromosome coordinates (x 10^{6})

B

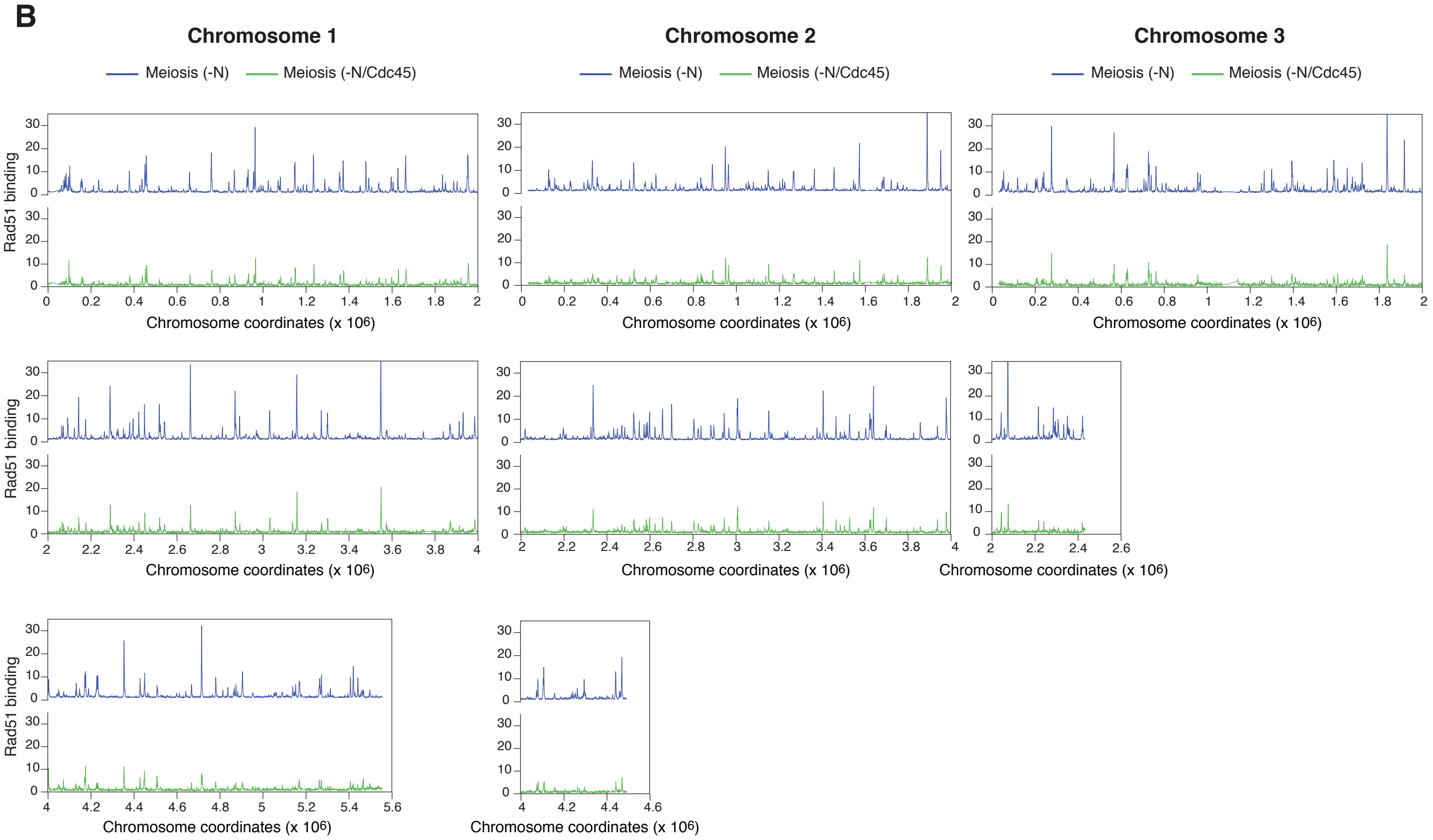

Chromosome 1
Chromosome 2
Chromosome 3
Meiosis (-N)
Meiosis (-N/Cdc45)
Rad51 binding
Chromosome coordinates (x 10^{6})

Figure S4 (related to Figure 3)

Modulation of Cdc45 levels alters origin efficiencies and induces specific changes in Rad51 binding

(A) Reduction in origin efficiencies by directly affecting the levels of Cdc45, a key pre-initiation complex component. Genome-wide representation of origin efficiencies in pre-meiotic S phase as determined by microarray analysis of copy number in two conditions: after nitrogen starvation (-N, blue) and after nitrogen starvation in strains with altered levels of Cdc45 (-N/Cdc45, green). In -N/Cdc45 conditions, overexpression of *cdc45* results in a prolongation of S phase linked to reduced origin usage across the genome. This likely occurs through a dominant-negative effect of high Cdc45 levels, which may titrate other critical limiting components of the machinery. The geometric mean of continuous windows of five probes was determined for each dataset before averaging dye-swap experiments (see Supplemental Experimental Procedures). The abscissa represents the moving average of the chromosome coordinates for the corresponding groups of probes. This figure displays a more detailed view of Fig. 3A.

(B) Rad51 binding to DNA in -N and -N/Cdc45 meiosis. Genome-wide representation of Rad51 binding during meiosis after nitrogen-depletion (-N, blue) or after nitrogen starvation in strains with altered levels of Cdc45 (-N/Cdc45, green). ChIP-on-chip analysis of Rad51 shows a greater number of peaks as well as greater peak heights in -N conditions compared to -N/Cdc45. Each dataset represents the average of two experiments; for each experiment, the geometric mean of continuous windows of five probes was first determined before averaging. The abscissa represents the moving average of the chromosome coordinates for the corresponding groups of probes, and the ordinate shows the level of Rad51 binding.

Table S1 (related to all Figures)

***Schizosaccharomyces pombe* strains used in this study.**

Strain	Genotype	Source
PN1	*h- 972*	Our stock
PN292	*h+ cdc25-22*	Our stock
PN2403	*h-/h- ade6-M210/ade6-M216*	Our stock
PN2159	*h+/h+ pat1-114/pat1-114 ade6-M210/ade6-M216*	Our stock
JW400	*h- cdc25-22 leu1-32 his7-336 leu1:adh1::hENT1 his7:adh1::tk*	Our stock
JW401	*h+/h+ pat1-114/pat1-114 ade6-M210/ade6-M216 leu1:adh1::hENT1/leu1:adh1::hENT1 his7-336/ his7-336 his7:adh1::tk/his7:adh1::tk*	This study
JW402	*h+/h+ his7-336/his7-336 leu1:adh1::hENT1/ leu1:adh1::hENT1 his7:adh1::tk/his7:adh1::tk*	This study
JW403	*h+/h+ pat1-114/pat1-114 ade6-M210/ade6-M216 msc1:kanMX6:rds1/+ SPAC3H1.10:natMX6:hsr1/+*	This study
JW404	*h+/h+ pat1-114/pat1-114 ade6-M210/ade6-M216 hrf1:kanMX6:egd2/+ SPBC3B8.04c:hphMX6:SPBC3B8.03/+*	This study
JW405	*h+/h+ pat1-114/pat1-114 ade6-M210/ade6-M216 SPBP8B7.32:ksnMX6:pop7/+ sat1:hphMX6:ssn6/+*	This study
JW406	*h+/h+ pat1-114/pat1-114 ade6-M210/ade6-M216 pma1:kanMX6:SPAC1071.13/+ SPAC683.03:natMX6:SPAC683.02c/+*	This study
JW407	*h+/h+ pat1-114/pat1-114 ade6-M210/ade6-M216 cta3:kanMX6:ibp1/+ SPBC119.05c:hphMX6:sco1/+*	This study
JW408	*h+/h+ pat1-114/pat1-114 ade6-M210/ade6-M216 SPBC36.02c:kanMX6:SPBC36.03c/+ ubi4:natMX6:erg28/+*	This study
JW409	*h+/h+ pat1-114/pat1-114 rec12Δ/rec12Δ*	This study
JW410	*h+/h+ pat1-114/pat1-114 nmt1-HA-Cdc45/nmt1-HA-Cdc45*	This study

Supplemental Experimental Procedures

Single-cell analysis of S phase length

To determine S phase length, we assumed that all cells in a given population take the same time to complete S phase. Thus, the first and last cells to begin DNA replication will also be the first and last to finish genome duplication, respectively. We used this latter population to determine S phase length by assessing the times at which these cells initiate and complete DNA synthesis. We first established when the last 10% of cells begins replication. For this, we engineered *pat1-114* diploids that can incorporate thymidine analogues into newly synthesized DNA (Sivakumar et al., 2004). These cells were induced to undergo synchronous meiosis in -N or +N conditions. EdU (Invitrogen) was added at the time of temperature shift (total labeling), and time points were taken at the indicated intervals (Fig. S1D). Cells were fixed in formaldehyde and permeabilized using Triton X-100, and EdU incorporation was assessed using the Click-iT EdU Imaging Kit (Invitrogen). The percentage of labeled cells at each time point was determined by microscopy ($n \geq 200$). As EdU is added prior to S phase onset, these curves represent the total percentage of cells that have already initiated or completed replication at any given point in the time course, reaching a plateau when all cells have entered S phase. The time interval at which the last 10% of cells enters S phase is shown in Fig. S1D (gray boxes on the black total labeling curves). Next, we assayed the percentage of cells still undergoing DNA synthesis at any given point. For this, we performed pulse labeling experiments, as this identifies cells actively replicating during the pulse. Similar experiments as above were conducted, except that EdU was only added to aliquots of the culture for 5 min at indicated times (Fig. S1D). The time interval at which the last 10% of cells finishes S phase is indicated (Fig. S1D, gray box on the gray pulse-labeling curves). The duration of S phase is estimated by the interval between the gray boxes (double-headed arrows). Similar values were obtained using the first 10% of cells to initiate and complete replication (data not shown).

Microarray data analysis

For origin efficiencies, the geometric mean over five probes was determined for each dye swap hybridization, and the datasets were averaged, reducing the impact of outliers. Analyses performed using the arithmetic mean yielded identical results. At least two biological repeats of each condition were performed, and representative experiments are shown (Fig. 1). Origins were identified using Microsoft Excel macros applying the following parameters: 1) a cutoff value of 1.1 for the copy number ratio (HU vs. unreplicated), similar to (Heichinger et al., 2006); 2) each peak value must be surrounded by 8 lower values on each side. This identifies origins that are a minimum of ~2kb apart. This selection was validated by visual analysis of the data and is supported by previous estimates that the extent of replication surrounding an origin in HU can reach 10-15 kb (Heichinger et al., 2006). For analysis and comparison of origin efficiencies in Figs. 2-4 and in Figs. S1C, S3A, and S3D, the baselines between different conditions, representing unreplicated DNA, were matched. To compare origin numbers, efficiencies, and positions between two experiments, peaks identified in each dataset were matched in position. As the microarray probes are only ~250 bp apart, the precise positions of the same peaks can vary slightly between experiments. Therefore, when peaks in two different datasets were less than 8 probes apart, the position of the +N peak was also assigned to the -N peak, consistent with the extent of replication in HU. For the differences in origin efficiencies shown in Fig. 2, the sums of the matched peak efficiencies over continuous ~200 kb windows were determined for each sample, the values were subtracted and plotted against the moving average of the corresponding chromosome coordinates.

For Rad51 binding, the geometric mean over five probes of each sample was determined, after which data from two biological repeats were averaged (Fig. S2B). To identify peaks of Rad51 binding, the following parameters were used: 1) a cutoff value of 1.5 for the ratio of IP vs. input; 2) each peak value must be surrounded by 8 lower values on each side. This 8-probe cutoff was validated by visual inspection of the Rad51 binding profiles. For comparisons of Rad51 binding, the same peak matching protocol as for origins was applied (see above). For Rad51 peak identification

and amplitude comparisons, the baselines of the binding profiles in the different conditions were matched (Figs. 2 and 3, Figs. S2C and S3A). To determine differences in the level of Rad51 binding, the sums of the matched peak values for continuous ~200 kb windows were determined for each sample, subtracted, and plotted against the moving average of the corresponding chromosome coordinates (Fig. S3A). For identification of changes in Rad51 binding in -N vs. +N (Fig. S2C), we identified sites where 1) a peak was unique to either condition or 2) the value of the peak showed a greater than two-fold difference in either condition. As we found very few unique or increased Rad51 binding sites in –N compared to +N (Fig. S2C), we subsequently focused only on the enrichment in +N. A density map showing increases in Rad51 binding in +N (Fig. 2A, bottom) was established by summing the number of selected peaks over continuous ~200 kb windows and plotting the data against the moving average of the corresponding chromosome coordinates. The Pearson's correlation coefficients for changes in origin efficiencies and density of changes in Rad51 binding (Fig. 2A) were calculated for each chromosome. Chromosome 1: 0.613 (99% confidence: 0.602-0.625); chromosome 2: 0.388 (99% confidence: 0.365-0.402); chromosome 3: 0.372 (99% confidence: 0.347-0.396). For chromosome 2, as there is only one major region that differs between the two parameters (the first 0.5 Mb of the left arm, representing ~10% of the chromosome), the correlation was calculated excluding this area, taking into account 90% of the entire chromosome. These correlation coefficients indicate a strong positive relationship between changes in origin efficiencies and changes in Rad51 binding in +N vs. -N conditions.

For comparison of origin efficiencies between -N and -N/Cdc45 (Fig. 3B), we modified the analysis described above. In both conditions, we found large areas of the genome in which active origins are either absent or below our detection threshold. Therefore, we focused our analysis on genomic regions in which we could clearly identify origins in both -N and -N/Cdc45. Furthermore, we took into account the lower origin efficiencies across the genome in -N/Cdc45 (Fig. S4A), setting the cutoff for origin identification in -N and -N/Cdc45 at 1.05 (copy number ratio, HU vs. unreplicated) and then confirming the selected peaks by visual screening of the microarray data.

For analysis of histone occupancy and modifications, we set the cutoff for peak selection for the histone H3 ChIP-on-Chip at 1.0 to be as permissive as possible; the cutoff for H3 K4-trimethylation was kept at 1.5, as for Rad51. Changes in H3 and H3 K4Me3 across the genome between -N and +N were determined as described above for Rad51.

Pulsed-field gel electrophoresis (PFGE)

Cells were treated as in Fig. S1A, and samples were collected after the shift to restrictive temperature at time points corresponding to those used for the genome-wide Rad51 binding experiments: 2h (+N, *pat1-114* and *pat1-114 rec12*Δ), 4h (-N, *pat1-114* and *pat1-rec12*Δ), 5h (-N, *pat1-114 nmt1-Cdc45*). The protocol used for the preparation of the DNA plugs is as described in (Ferreira and Cooper, 2001). Briefly, cells were digested with zymolyase and embedded in a low-melting agarose plug. The plug was treated with proteinase K for 48 hours, followed by incubation with PMSF. Digestion with NotI restriction enzyme was carried out at 37ºC overnight. DNA was separated on a Bio-Rad CHEF DRIII for 28 hours using the following parameters: 60-120 sec switch time, 120º angle, 6V/cm. Migrated DNA was visualized using ethidium bromide, and gels were transferred for Southern blotting and hybridized with a probe for the left end of NotI fragment L labeled using the Agilent Prime-It kit. A Storm PhosphorImager (Molecular Dynamics) was used for imaging of hybridized blots.

Supplemental References

Cromie, G.A., Hyppa, R.W., Cam, H.P., Farah, J.A., Grewal, S.I.S., and Smith, G.R. 2007. A discrete class of intergenic DNA dictates meiotic DNA break hotspots in fission yeast. *PLoS Genet* **3:**e141.

Ferreira, M.G., and Cooper, J.P. 2001. The fission yeast Taz1 protein protects chromosomes from Ku-dependent end-to-end fusions. *Mol Cell* **7:**55-63.

Heichinger, C., Penkett, C.J., Bahler, J., and Nurse, P. 2006. Genome-wide characterization of fission yeast DNA replication origins. *Embo J* **25:**5171-5179.

Hyppa, R.W., Cromie, G.A., and Smith, G.R. 2008. Indistinguishable landscapes of meiotic DNA breaks in rad50+ and rad50S strains of fission yeast revealed by a novel rad50+ recombination intermediate. *PLoS Genet* **4:**e1000267.

Sivakumar, S., Porter-Goff, M., Patel, P.K., Benoit, K., and Rhind, N. 2004. In vivo labeling of fission yeast DNA with thymidine and thymidine analogs. *Methods* **33:**213-219.

Tischfield, S.E., and Keeney, S. 2012. Scale matters: the spatial correlation of yeast meiotic DNA breaks with histone H3 trimethylation is driven largely by independent colocalization at promoters. *Cell Cycle* **11:**1496-1503.
